# Difelikefalin in the Treatment of Chronic Kidney Disease-Associated Pruritus: A Systematic Review

**DOI:** 10.3390/ph15080934

**Published:** 2022-07-28

**Authors:** Kamila Wala, Jacek C. Szepietowski

**Affiliations:** Department of Dermatology, Venereology and Allergology, Wroclaw Medical University, 50-368 Wroclaw, Poland; kamila.wala.01@gmail.com

**Keywords:** difelikefalin, kappa-opioid receptor, chronic kidney disease, pruritus, hemodialysis, itch

## Abstract

Chronic kidney disease-associated pruritus (CKD-aP) is a chronic condition that significantly reduces the quality of life of patients with end-stage renal disease. The etiology is not fully understood, but imbalance in the activity of the opioid pathways, including downregulation of the kappa-opioid receptor, may contribute to itching sensation. Difelikefalin is a selective, peripherally acting kappa-opioid receptor (KOR) agonist. Recently, difelikefalin has been approved as a first drug for the treatment of pruritus associated with chronic kidney disease (CKD) in adult hemodialysis patients. A systematic review of currently available clinical trials was performed to assess the efficacy and safety of difelikefalin in patients with uremic pruritus. A literature review was conducted in May 2022 based on the PRISMA 2020 guidelines. The analyzed clinical trials showed that difelikefalin was effective in reducing pruritus in patients as assessed by the Worst Itching Intensity Numerical Rating Scale. Improvement in quality of life assessed on the basis of the Skindex score and the 5-D itch scale was also noticed. The most commonly reported side effects were mild and included nausea, vomiting, dizziness, and diarrhea. Due to its proven efficacy and good safety profile, difelikefalin is a promising drug for the treatment of pruritus in patients with chronic kidney disease.

## 1. Introduction

Chronic kidney disease-associated pruritus (CKD-aP), also known as uremic pruritus, is a condition that significantly reduces the quality of life of patients with end-stage renal disease [1]. Persistent pruritus negatively affects both physical and mental health. Patients with CKD-aP suffer from sleep disorders, depression, and also have an increased risk of cardiovascular diseases [2]. In addition, constant scratching of the skin contributes to the formation of secondary changes, including bacterial infections [3].

The prevalence of CKD-aP reported in the literature ranges from 20 to 80% and varies depending on the stage of the kidney disease and the type of dialysis used (hemodialysis or peritoneal dialysis) [4]. Based on current studies, it is estimated that moderate to severe pruritus occurs in approximately 40% of patients with chronic kidney disease (CKD) [5]. The etiology is not fully understood, but there are many hypotheses that may contribute to the onset and severity of CKD-aP. Among others, mediators such as histamine or serotonin, dysregulation of the activity of opioid pathways (including kappa-opioid receptor (KOR) downregulation), xerosis, hyperparathyroidism, accumulation of uremic toxins, and dysregulation of the immune system are suspected to be significant factors of chronic itch [4,6,7].

Despite the many different therapeutic options for treating pruritus, CKD-aP remains a significant therapeutic challenge. The treatment of CKD-aP with commonly used antihistamines has been shown to be ineffective in this group of patients [8,9]. Drugs that act on the central nervous system (CNS), such as gabapentin or pregabalin, appear to be of greatest importance among oral medications in the treatment of this type of pruritus. However, systemic side effects, such as dizziness, somnolence, loss of balance, and fatigue, have been noted [10]. In clinical trials by Seckin et al. [11] and Gilchrest et al. [12] with ultraviolet (UV) B therapy in patients with CKD-aP, significant improvement in pruritus severity was observed, with the most common adverse reaction being mild local sunburn. On the other hand, in alternative studies by Hsu et al. [13] and Ko et al. [14], after narrowband UVB application, no side effects were noticed, but also no statistically significant improvement was shown compared to the control group. It is worth mentioning that studies on the effects of UVB phototherapy in patients with CKD-aP were conducted on small groups of patients, and the control group and possible placebo effect were not taken into account [13,14]. Topical treatment, due to the patient’s good tolerance, can be used in CKD-aP patients; however, it is often insufficient in monotherapy. In turn, emollients should be included in any treatment regimen, regardless of the use of additional therapy [15]. Despite the increasing awareness of the importance of the CKD-aP problem, this condition is underdiagnosed, and some patients still do not receive sufficient treatment for chronic pruritus.

In recent years, many studies have focused on understanding the correlation between an imbalance in opioid signaling and the incidence of pruritus. Activation of mu-opioid receptors (MOR) in analgesia (e.g., by morphine) has been shown to cause an itching sensation [16]. Furthermore, it has been noticed that lowering the KOR level and increased activation of MOR occurs in patients with chronic pruritus, including CKD-aP [15,17]. In clinical trials and in case reports, the antipruritic effect of substances belonging to MOR antagonists (MORA) has been demonstrated. A beneficial effect of MORA, especially in the treatment of cholestatic pruritus, has been noted; however, the data on the effectiveness of this substances in the treatment of CKD-aP are divergent [18,19,20,21,22]. On the other hand, the activation of opioid pathways by acting on KOR has strong antipruritic properties [23]. KOR agonists, such as nalbuphine, nalfurafine, and ZYKR1, have been shown to be effective in preclinical animal studies, in which these compounds reduce scratching caused by variable pruritogens [24,25,26,27]. Moreover, no similar antipruritic effect was observed after administration of nalbuphine in mice lacking the KORs, which confirms the mechanism of action of this substance through activation of this receptors [26]. For nalfurafine, in addition to favorably inhibiting scratching in male rats, the addictive potential of this agonist was reported [24]. However, no psychological or physical dependence was demonstrated in preclinical studies in Rhesus monkeys or during 52 weeks of follow-up in clinical trials [28,29,30]. In randomized, placebo-controlled clinical trials of the KOR agonists nalfurafine and nalbuphine, statistically significant reductions in pruritus were reported in patients with CKD-aP and prurigo nodularis, respectively [31,32].

**Figure 1:**
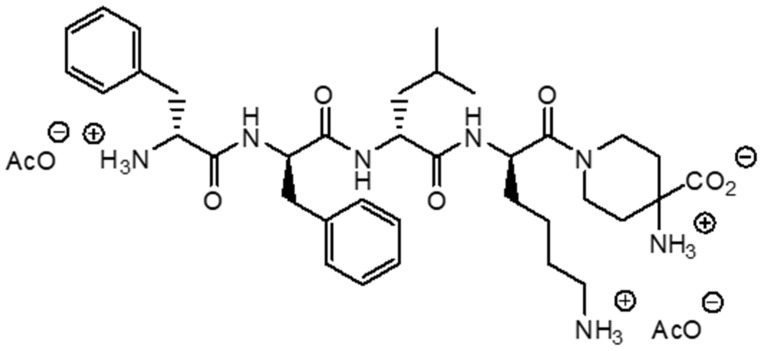
Chemical structure of difelikefalin.

Difelikefalin is a novel opioid agonist with high selectivity for KOR that has been shown to be effective in the treatment of chronic pruritus and post operative pain [33]. By activating KOR on peripheral sensory neurons, it inhibits the afferent transmission of sensory signals to the CNS. Furthermore, this drug has an immunomodulatory effect. It has been shown to activate KORs on cells of the immune system, leading to a reduction in the production of pro-inflammatory cytokines and a reduction in inflammation [23,34]. Importantly, with hydrophilic properties, its transport across the blood–brain barrier is limited. Compared to many other opioids, such as fentanyl or morphine, difelikefalin exhibits a minimal effect on the central nervous system and does not cause, among others, respiratory depression or sedation [33]. In a randomized clinical trial, Viscusi et al. [35] showed that difelikefalin administered intravenously at a dose of 1.0 and 5.0 µg/kg did not cause respiratory depression, and all the noted side effects, such as somnolence or paresthesia, were mild and did not require any intervention. Due to the lack of influence on MOR, no euphoric effect was observed when using difelikefalin; therefore, the addictive potential of this substance is low [36]. In August 2021, difelikefalin was approved by the Food and Drug Administration (FDA) and in April 2022 by European Medicines Agency (EMA) as a first drug for the treatment of CKD-aP in adult, hemodialysis patients. The recommended dose is 0.5 μg/kg body weight. The drug is administered as an intravenous injection at the end of hemodialysis session [37]. The structure of difelikefalin is shown in Figure 1.

The aim of this study is to evaluate the clinical efficacy and safety as well as to summarize the current knowledge of difelikefalin in treatment of patients with CKD-aP based on the available clinical trials.

## 2. Methods

The literature review was conducted independently by two authors in May 2022 based on the PRISMA 2020 guideline [38]. The systematic review has been registered in International Platform of Registered Systematic Review and Marta-Analysis Protocols–INPLASY (registration number: INPLASY202250154). The PubMed, ScienceDirect, and Scholar Google databases were searched for relevant articles using the combination of the keywords “difelikefalin” or “CR845” AND “pruritus” or “itch” or “chronic kidney disease” or “hemodialysis”. Initially, 457 articles were found. Firstly, after excluding duplicate records, 375 works were included in the further screening.

**Figure 2:**
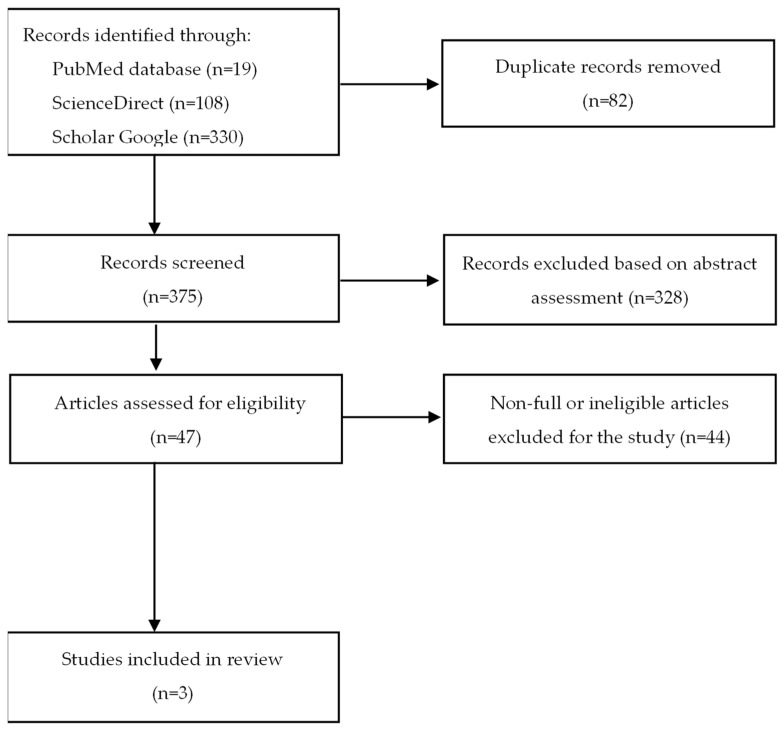
Flow diagram of the literature search procedure.

Then, a gradual selection was made, as shown in Figure 2. The flow diagram was created based on the PRISMA template [38]. The exclusion criteria included all types of articles except for research works (e.g., reviews, letters) and a language other than English. Inclusion criteria were full-text original articles on the effects of difelikefalin in dialysis on adult patients with chronic pruritus. Based on abstracts, unrelated topics, review articles, and letters were rejected. In addition, articles written in a language other than English were not eligible for further scanning. The remaining studies were then analyzed. At the final stage, incomplete and ineligible articles were excluded. Eventually, three articles were included in the work.

In addition, the website of the National Institute of Health was searched for information related to new clinical trials related to the topic.

**Figure 3:**
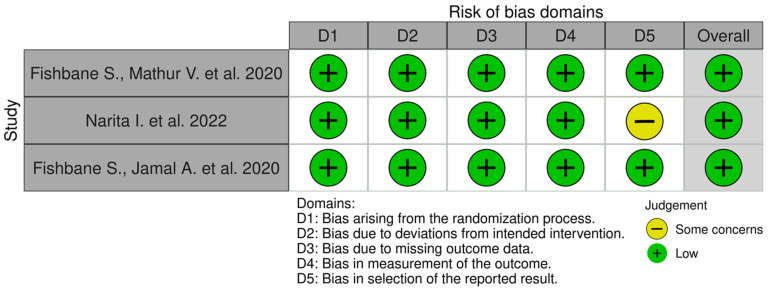
Risk of bias. The Robvis tool was used to create the risk-of-bias plots [39,40,41,42].

Selected publications were carefully analyzed independently by two authors for possible deviations in the results, which could result from inappropriate selection of participants, randomization, or evaluation of the results. The included clinical trials passed the randomization process and had a low overall risk of misleading results. The criteria for qualifying patients to participate in the study were defined in detail. The research was carried out according to the protocol. The results of both studies by Fishbane et al. [39,40] have been well presented, both the baseline data and the data, at the end of the entire study. Narita et al. [41] described the results, but not every value was presented—some results from the placebo groups were missing from the main text. However, all data from the difelikefalin and placebo groups are presented in the tables attached to the article. The overall risk of misleading results was assessed as low. The risk of bias for individual domains in analyzed clinical trials is presented in Figure 3.

## 3. Results

Until May 2022, three clinical trials evaluating the effectiveness of difelikefalin in the treatment of CKD-aP have been published, in which a total of 800 patients with moderate to severe CKD-aP participated [39,40,41]. Patients assessed the intensity of symptoms before, during, and after the trial.

In a randomized, double-blind, placebo-controlled phase 2 study conducted in the United States (NCT02858726), participants (*n* = 174) were divided into a total of four groups and received placebo or difelikefalin at different doses: 0.5 μg/kg (*n* = 44), 1.0 μg/kg (*n* = 41), or 1.5 μg/kg (*n* = 44). The substances were administered intravenously after each hemodialysis session 3 times a week for 8 weeks. Participants were asked to complete the questionnaires: WI-NRS (Worst Itching Intensity Numerical Rating Scale); Skindex-10; 5-D itch scale; Medical Outcomes Study sleep disturbance subscale; Patient Global Impression of Worst Itch Severity; and Patient Global Impression of Change [39]. Another randomized, multicenter, double-blind, placebo-controlled phase 2 trial included 247 participants from Japan with moderate to severe pruritus (NCT03802617). Patients were administered placebo or difelikefalin at a dose of 0.25 μg/kg, 0.5 μg/kg or 1.0 μg/kg in the form of intravenous boluses. As in the previous study, the effect of difelikefalin was based on the changes in the weekly mean NRS score. Secondary outcomes included Skindex-16 and the 5-D itch scale [41]. In turn, in a randomized, double-blind, placebo-controlled phase 3 trial (NCT03422653, KALM-1), all participants (*n* = 378) were divided into two groups: the placebo group (*n* = 165) and the group receiving difelikefalin at a dose of 0.5 µg/kg (*n* = 158). Patients were administered either the drug or placebo injection at the end of the hemodialysis session, 3 times per week for 12 weeks. Then, after finishing the treatment protocol, patients were observed for a further 2 weeks to exclude any addictive effect of opioid drug. The results were assessed using the following scales: WI-NRS, Skindex-10, and 5-D itch scale. The safety of difelikefalin in all clinical trials was evaluated on the basis of laboratory tests, vital signs, a 12-lead electrocardiogram, and patient-reported side effects occurring throughout the clinical trial [40].

### 3.1. Evaluation of Effectiveness of Difelikefalin

The effectiveness of the new drug was evaluated on the basis of the changes in pruritus severity reported by the patients and the assessment of the impact of pruritus on quality of life in its various aspects, including sleep quality.

#### 3.1.1. Effect of Difelikefalin on Pruritus Severity

In all the analyzed studies, difelikefalin significantly reduced the severity of pruritus compared to the control group receiving placebo. In a phase 2 clinical trial among patients receiving difelikefalin, there was a reduction in itching severity of −3.8, −2.8, and −3.2 for a dose of 0.5 µg/kg, 1.0 µg/kg, and 1.5 µg/kg, respectively. The difference was statistically significant in the 0.5 µg/kg (*p* < 0.001) and 1.5 µg/kg (*p* = 0.019) difelikefalin groups. Furthermore, after 8 weeks of treatment, a depletion in pruritus of at least 3 points from baseline on the WI-NRS was reported in 59–64% of patients in groups receiving difelikefalin compared to 29% in the placebo group [39]. In clinical trial conducted in Japan Narita et al. [41] reported a significant reduction in itching on the NRS score at week 8 in the difelikefalin 0.5 and 1.0 µg/kg group (−3.65 and −3.64, respectively) compared to the placebo group (−2.86). An improvement of 3 points from the mean weekly NRS score was seen in 53%, 60%, and 57% for difelikefalin concentrations of 0.25, 0.5, and 1.0 µg/kg, respectively, compared to 50% in the placebo group. A 4-point reduction in the pruritus score occurred in 36% of patients in the placebo group, 34% in the difelikefalin 0.25 μg/kg group, 51% in the difelikefalin 0.5 μg/kg group, and 43% in the difelikefalin 1.0 μg/kg. Only for the 0.5 and 1.0 µg/kg groups was the difference statistically significant [41]. In contrast, in a phase 3 clinical trial from Fishbane et al. [40], the reduction in WI-NRS of 3 or more points after 12 weeks was observed in 49.1% and 27.9% of patients in the difelikefalin and placebo groups, respectively. In turn, an improvement from baseline of at least 4 points on the WI-NRS scale was reported in 37.1% of patients treated with difelikefalin and 17.9% of participants in the control group.

#### 3.1.2. Effect of Difelikefalin on Itch-Related Quality of Life

Difelikefalin has also been shown to be effective in improving itch-related quality of life. At week 8, the mean total Skindex-10 score reported by Fishbane et al. [39] changed by −16.4 points in the difelikefalin groups and the decrease was twice as high as in placebo group, where the score diminished by −8.2 points (*p* < 0.001). A similar improvement was seen on the 5-D itch scale with a reduction ranging from 4.7 to 5.7 points at week 8 in the difelikefalin groups compared to 2.8 points in the placebo group (*p* < 0.001). After 8 weeks of the Japanese clinical trial, an improvement in the 5-D itch scale total score and Skindex-16 overall score was also shown in all the studied groups [41]. However, the weekly mean reduction of the Skindex-16 overall score differs only for difelikefalin at the dose of 0.5 μg/kg from the placebo group (−27.79 points versus the placebo at −24.04 points). Interestingly, in the final Patient Global Impression of Change at the end of the treatment period, answers “very much improved” or “much improved” were reported by 26 (41.9%) patients in the placebo group. The same degrees of improvement were significantly more frequently marked by participants from the difelikefalin groups (33 (54.1%), 39 (66.1%), and 42 (70%) at doses of 0.25 μg/kg, 0.5 μg/kg, and 1.0 μg/kg, respectively) [41]. In phase 3, after administration of difelikefalin (0.5 μg/kg) at week 12, patients improved from baseline on the Skindex-10 scale by −17.2 ± 1.3 points and on the 5-D itch scale by −5.0 ± 0.3 points. In the placebo group, the mean changes were −12.0 ± 1.2 and −3.7 ± 0.3 points, respectively. The differences were statistically significant with *p*-values less than 0.001. An equally important disturbance aspect in patients with chronic pruritus is sleep quality. Patients in all difelikefalin groups reported an improvement in Itch MOS sleep disturbance by an average of −11.8 ± 2.0 points (*p* = 0.005). In the placebo group, the difference was much less pronounced, at −1.3 ± 3.1 points from the baseline value [39].

### 3.2. Adverse Effects

The most common adverse effect in all clinical trials was diarrhea, nausea, vomiting, dizziness, fall, and headache. They were usually mild to moderate in intensity. The more severe side effects included somnolence, other mental status changes, hypotension, pneumonia, and sepsis [39,40,41]. There were also four deaths due to septic shock during the phase 3 studies, but with equal frequency in the placebo group and with difelikefalin 0.5 µg/kg [40]. In a second phase clinical trial by Narita et al. [41] an enhancement in the incidence of side effects has been noticed when increasing the difelikefalin dose. However, a similar dependence did not occur in the study from Fishbane et al. [39], which also investigated different doses of the drug. Nevertheless, in each analyzed study, side effects were more frequently observed after injection of difelikefalin compared to the placebo. Interestingly, in a phase 3 trial, more severe adverse events were similarly common in the placebo and 0.5 μg/kg difelikefalin group [40]. Additionally, after the end of the treatment, no symptoms of physical or psychological dependence on the administered drug were noticed during the two-week observation.

The summary of the data from the analyzed clinical trials is presented in Table 1.

## 4. Discussion

Pruritus is defined as an unpleasant sensation that makes you scratch. It is a common symptom of many dermatological diseases, but it can also be associated with systemic disorders, such as liver and chronic kidney diseases, or some neoplastic diseases [43]. In the case of advanced chronic renal failure (stages 3–5), the average incidence is about 45% [44]. Importantly, 18% of dialysis patients say that the severity of itch is “very much” or “extreme”, confirming the importance of the problem of pruritus in patients with end-stage CKD and the need to improve pruritus treatment [45].

In the studies analyzed in this review, several scales were used to assess the severity of itching, which allowed to assess the effectiveness of difelikefalin and to compare the results of various studies with each other. Itching is a subjective sensation reported by patients. Many scales have been developed to standardize this symptom and compare the severity of pruritus and its impact on different domains of life in different patient groups, as well as changes in severity over time in the same patient. The primary methods include the itch severity assessment with Numerical Rating Scale (NRS) in which the patient rates itch from 1 point (no itch) to 10 points (worst imaginable pruritus). Based on the score, pruritus is divided into mild (1–3 points), moderate (4–6 points), severe (7–8 points), and very severe (9 points or more) [46]. WI-NRS determines the worst possible itching intensity that patients have experienced. It has been shown to be very easy to use and exhibits a high accuracy and reliability in patients with CKD-aP, as well as with psoriasis or nodular pruritus [47,48,49]. Therefore, this scale is very willingly used in clinical trials. In the publications analyzed in this review, the authors used WI-NRS to assess the severity of baseline pruritus before treatment and the change in pruritus severity after administration of the study drug. The results are presented as the weekly average of the reported WI-NRS score. Additionally, the percentage of people who improved on this scale by at least 3 or 4 points was compared. However, a single assessment of the severity of itch only does not reflect the significance of the problem of chronic pruritus. Therefore, an additional multi-directional pruritus assessment tool has been developed [7]. The 5-D itch scale (five dimensions: degree, duration, direction, disability, and distribution) assesses not only the severity of the itching but also the duration, changes over time, and its impact on sleep and daily functioning. This scale has been shown to be reliable and appropriate for assessing pruritus in patients with dermatological diseases as well as in patients with chronic kidney disease [50]. Other tools to assess health-related QoL include the multidimensional Skindex-10 and the MOS Sleep Disorder subscale, which again demonstrate high reliability and validity in hemodialysis patients with CKD-aP [51]. Moreover, in a study of 2323 participants with chronic pruritus, individual scales (itch NRS, WI-NRS, and 5-D itch scale) show a significant positive correlation and can be effectively used simultaneously to assess chronic pruritus intensity in clinical trials [52]. Fishbane et al. [39] also emphasized that the reduction in the weekly average of the WI-NRS score after difelikefalin or placebo administration is highly correlated with an improvement in the itch 5-D total score and Skindex-10 score, where the Pearson’s correlation coefficient (r) was set at 0.71 and 0.67, respectively [39].

In recent years, numerous clinical trials have been conducted to evaluate the efficacy and safety of difelikefalin in patients with persistent pruritus. Most of them are related to CKD-aP. A total of 13 clinical trials have been performed or are currently ongoing in patients with CKD undergoing hemodialysis [53,54,55,56,57,58,59,60,61,62,63,64]. The effect of this drug in reducing itching in patients with cholestatic or atopic dermatitis-related pruritus is also being investigated [65,66]. Table 2 presents a summary of clinical trials along with their brief characteristics.

In the clinical trials analyzed in this review, difelikefalin was administered by intravenous injection at doses of 0.25–1.5 µg/kg three times per week on dialysis days. The available research results from a phase 2 clinical trial show no statistically significant improvement with difelikefalin at a dose of 0.25 µg/kg compared to the placebo group. In turn, the effectiveness of difelikefalin at a dose of 0.5, 1.0, and 1.5 µg/kg was comparable in both studies of the second phase. No explicit results indicating an enhancement in the frequency of side effects with increasing doses of the drug have been seen. Incidence of side effects in dose-dependent manner was found in only one analyzed study [41]. However, Fishbane et al. [39] reported a similar frequency of adverse events in all difelikefalin groups. In turn, in a randomized, double-blinded, placebo-controlled trial in healthy volunteers, difelikefalin in doses up to 4 µg/kg was well tolerated. The incidence of side effects such as paresthaesia, dizziness, fatigue, or nausea was dose dependent, but even at the highest dose these events were mild in intensity [68]. Studies in CKD-aP patients have not shown any superiority in the use of difelikefalin at doses greater than 0.5 µg/kg, with a comparable or even greater risk of side effects. Therefore, in phase 3 clinical trials, the 0.5 µg/kg dose of difelikefalin is most commonly used to further evaluate the efficacy and safety of this drug in a larger population and over a longer period of time [40,55,56,59].

Despite the fact that some studies are completed, their results have not been published so far. Nevertheless, a few studies are available in the form of conference abstracts. This applies, among others, to the KALM-2 study, the results of which were important for the approval of difelikefalin by the FDA for clinical use [69]. The published results of phase 2 and 3 studies have already confirmed both the efficacy and safety of difelikefalin in patients with end-stage renal disease undergoing hemodialysis. However, the duration of the studies analyzed was a maximum of 12 weeks [39,40,41]. In turn, a randomized, multicenter, placebo-controlled phase 3 study (KALM-2) enrolled 473 participants and showed good tolerance with an acceptable long-term safety profile during the 52-week study [69]. In August 2021, the FDA approved the use of difelikefalin (under the trade name Korsuva) for the treatment of CKD-aP in adult hemodialysis patients. Thus, this drug became the first substance specifically indicated for the treatment of uremic pruritus [37]. Subsequently, in April 2022, EMA also approved the use of difelikefalin (trade name: Kapruvia) for the treatment of moderate to severe pruritus in dialysis patients in EU countries [70]. Both the FDA and EMA have approved the use of difelikefalin at a dose of 0.5 µg/kg dry weight administered as an intravenous injection at the end of a hemodialysis session. However, the results of ongoing studies evaluating the efficacy of oral administration of difelikefalin at doses of 0.25, 0.5, 1.0, or 2.0 mg per day in the treatment of CKD-aP are also awaited [60,63,64]. Importantly, with the documented efficacy and safety of the oral form of the drug, this method of administering difelikefalin would be much more favorable in patients with CKD-aP, but not requiring dialysis.

The most common side effects of difelikefalin reported in clinical trials were mild and included nausea, vomiting, diarrhea, dizziness, and headache. In the phase 3 of the clinical trial, no symptoms of drug addiction were observed for 2 weeks of follow-up after the end of treatment [40]. Interestingly, another study also showed that even many times higher doses (5 and 15 µg/kg) of difelikefalin have a low addictive potential [36]. Moreover, unlike other opioid drugs, such as nalbuphine or nalfurafine, difelikefalin has limited penetration into the CNS. In a phase 3 clinical trial with nalfurafine, a centrally acting selective KOR activator, insomnia was observed much more often compared to the difelikefalin trials, and was the most frequently reported symptom [32]. None of the analyzed studies in this article reported a depressive effect of difelikefalin on the respiratory system, which is major limitation in the use of centrally acting opioids [39,40,41]. Therefore, difelikefalin, as new drug that selectively acts on peripheral KOR, has a more favorable safety profile compared to the other opioid agents. In clinical trials investigating the analgesic efficacy and safety profile of difelikefalin at a dose of 5 µg/kg, the most common side effects were headache and dizziness; no serious side effects were observed [71]. Importantly, in studies on the effectiveness of difelikefalin in patients with CKD-aP, even after the administration of a low dose of 0.5 µg/kg, statistically significant better results were obtained compared to the placebo group. As mentioned above, higher doses of difelikefalin (up to 1.5 µg/kg) did not show any advantage over the dose of 0.5 µg/kg. This may be the reason why there are no clinical trials testing this drug at much higher doses; for example, those used to relieve pain.

### Limitation of the Study

A significant limitation of this systematic review was the lack of access to the results of completed trials; however, these are not yet published. In addition, the interpretation and comparison of the results of studies conducted in different countries, i.e., among other populations with different access to treatment (for example, the use of nalfurafine in the treatment of CKD-aP only in Japan, or different preferences for dialysis methods used), may lead to misleading conclusions. Furthermore, data on the efficacy of difelikefalin in the treatment of CKD-aP are limited to a small dose range (0.25–1.5 µg/kg). The efficacy of difelikefalin at higher doses in patients with CKD-aP has not been analyzed.

## 5. Conclusions

Our systematic review shows that difelikefalin, due to its efficacy and good safety profile, can be regarded as the primary treatment for pruritus in patients with chronic kidney disease. Research on this subject should be continued to evaluate the long-term effects of difelikefalin administration.

## Figures and Tables

**Table 1:** Summary of the analyzed clinical trials data.

Title	Fishbane et al. [39]	Narita et al. [41]	Fishbane et al. [40]
Phase	2	2	3
Country	US	Japan	US
Number of patients	175	247	378
Time to end-point	8 weeks	8 weeks	12 weeks
Reserach groups	Placebo (*n* = 45) Difelikefalin 0.5 μg/kg (*n* = 44) Difelikefalin 1.0 μg/kg (*n* = 42) Difelikefalin 1.5 μg/kg (*n* = 44)	Placebo (*n* = 63) Difelikefalin 0.25 μg/kg (*n* = 61) Difelikefalin 0.5 μg/kg (*n* = 61) Difelikefalin 1.0 μg/kg (*n* = 62)	Placebo (*n* = 165) Difelikefalin 0.5 μg/kg (*n* = 158)
Evaluated parameters	WI-NRS Skindex-10 5-D itch scale Medical Outcomes Study sleep disturbance subscale Patient Global Impression of Worst Itch Severity Patient Global Impression of Change	WI-NRS Skindex-16 5-D itch scale Patient Global Impression of Change	WI-NRS Skindex-10 5-D itch scale
Changes from baseline in WI-NRS			
-reduction of weekly mean WI-NRS	Placebo group: −1.9 Difelikefalin 0.5 μg/kg: −3.8 Difelikefalin 1.0 μg/kg: −2.8 Difelikefalin 1.5 μg/kg: −3.2	Placebo group: −2.86, Difelikefalin 0.25 μg/kg: −2.97 Difelikefalin 0.5 μg/kg: −3.65 Difelikefalin 1.0 μg/kg: −3.64	N/A
-reduction of at least 3 points	Placebo group: 29% Difelikefalin 0.5 μg/kg: 64% Difelikefalin 1.0 μg/kg: N/A Difelikefalin 1.5 μg/kg: 67%	Placebo group: 50% Difelikefalin 0.25 μg/kg: 53% Difelikefalin 0.5 μg/kg: 60% Difelikefalin 1.0 μg/kg: 57%	Placebo group: 27.9% Difelikefalin 0.5 μg/kg: 49.1%
-reduction of at least 4 points	Placebo group: 24% Difelikefalin 0.5 μg/kg: 51% Difelikefalin 1.0 μg/kg: N/A Difelikefalin 1.5 μg/kg: N/A	Placebo group: 36%, Difelikefalin 0.25 μg/kg: 34% Difelikefalin 0.5 μg/kg: 51% Difelikefalin 1.0 μg/kg: 43%	Placebo group: 21.2% Difelikefalin 0.5 μg/kg: 40.5%
Changes from baseline after treatment in Skindex-10 or Skindex-16 (points)	Placebo group: −8.2 Difelikefalin 0.5 μg/kg: −18.7 Difelikefalin 1.0 μg/kg: −15.5 Difelikefalin 1.5 μg/kg: −15.1	* Placebo group: −24.04 * Difelikefalin 0.25 μg/kg: −24.25 * Difelikefalin 0.5 μg/kg: −27.79 * Difelikefalin 1.0 μg/kg: −22.69	Placebo group: –12.0 Difelikefalin 0.5 μg/kg: −17.2
Changes from baseline after treatment in 5-D itch scale (points)	Placebo group: −2.8 Difelikefalin 0.5 μg/kg: −5.7 Difelikefalin 1.0 μg/kg: −5.4 Difelikefalin 1.5 μg/kg: −4.7	Placebo group: −5.8 Difelikefalin 0.25 μg/kg: −6.6 Difelikefalin 0.5 μg/kg: −6.5 Difelikefalin 1.0 μg/kg: −6.8	Placebo group: –3.7 Difelikefalin 0.5 μg/kg: –5.0
Adverse effects			
-overall incidence	Placebo group: 4 patients (8.9%) Difelikefalin 0.5 μg/kg: 10 patients (22.7%) Difelikefalin 1.0 μg/kg: 6 patients (14.6%) Difelikefalin 1.5 μg/kg: 11 patients (25.0%)	Placebo group: 42 patients (67%) Difelikefalin 0.25 μg/kg: 44 patients (72%), Difelikefalin 0.5 μg/kg: 47 of 61 patients (77%), Difelikefalin 1.0 μg/kg: 53 of 62 patients (85%)	Placebo group: 117 patients (62.2%) Difelikefalin 0.5 μg/kg: 130 patients (68.8%)
-symptoms	Mild: diarrhea, dizziness, nausea, fall, headache More severe: somnolence, abdominal pain, mental status changes	Mild: dizziness, vomiting, nasopharyngitis More severe: somnolence, hypotension	Mild: diarrhea, dizziness, vomiting More severe: hyperkalemia, pneumonia, sepsis, hypotension and chronic obstructive pulmonary disease

(N/A–not available; *—Skindex-16 score).

**Table 2:** Clinical trials of difelikefalin conducted in patients with pruritus (N/A—not available; table created on the basis of data available on the website https://www.clinicaltrials.gov/ [67]).

Title	ClinicalTrials.Gov Identifier	Condition	Phase	Status	Number of Participants	Dose of Difelikefalin	Year
Safety and Pharmacokinetics of IV CR845 in Hemodialysis Patients, and Its Efficacy in Patients With Uremic Pruritus	NCT02229929	CKD-aP	2	Completed	89	0.5 μg, 1.0 μg, or 2.5 μg/kg administered after each dialysis session over a 1 week treatment period (3 times/week)	2014–2016
Study to Evaluate IV CR845 in Hemodialysis Patients With Moderate-to-Severe Pruritus	NCT02858726	CKD-aP	2/3	Completed	226	0.5 μg, 1.0 μg, or 1.5 μg/kg administered after each dialysis session (3 times/week)	2016–2018
Extension Study to Evaluate IV CR845 in Hemodialysis Patients With Moderate-to-Severe Pruritus	NCT03281538	CKD-aP	3	Completed	288	0.5 μg/kg administered after each dialysis session (3 times/week)	2017–2021
A Study to Evaluate the Safety and Efficacy of CR845 in Chronic Kidney Disease Patients With Moderate-to-Severe Pruritus	NCT03617536	CKD-aP	2	Completed	271	0.25, 0.5 or 1 mg; oral; once a day	2018–2020
A Study to Evaluate the Safety and Efficacy of CR845 in Hemodialysis Patients With Moderate-to-Severe Pruritus (KALM-1)	NCT03422653	CKD-aP	3	Completed	378	0.5 μg/kg administered after each dialysis session (3 times/week)	2018–2020
CR845-CLIN3103: A Global Study to Evaluate the Safety and Efficacy of CR845 in Hemodialysis Patients With Moderate-to-Severe Pruritus	NCT03636269	CKD-aP	3	Completed	474	0.5 μg/kg administered after each dialysis session (3 times/week)	2018–2021
CR845-CLIN3105: A Study to Evaluate the Safety and Effectiveness of CR845 in Hemodialysis Patients With Moderate-to-Severe Pruritus	NCT03998163	CKD-aP	3	Completed	222	0.5 μg/kg administered after each dialysis session (3 times/week)	2019–2021
A Clinical Study of MR13A9 in Hemodialysis Patients With Pruritus	NCT03802617	CKD-aP	2	Completed	247	0.25 mg, 0.5 mg, or 1.0 mg/kg administered after each dialysis session (3 times/week)	2019–2019
Study to Evaluate the Pharmacokinetics and Metabolism of [14C] CR845 (Difelikefalin) in Patients With End Stage Renal Disease on Hemodialysis and in Healthy Subjects	NCT03947970	Healthy and hemodialysis patients	1	Completed	12	intravenous bolus-the total dose of CR845 will range from 1.7 to 3.1 μg/kg	2019–2019
Intermediate-Size Patient Population Expanded Access Program for Intravenous Difelikefalin	NCT05031546	CKD-aP	N/A	Available	N/A Expanded Access to Treatmend	0.5 μg kg administered after each dialysis session (3 times/week)	2021-
A Phase III Clinical Study of MR13A9 in Hemodialysis Patients With Pruritus	NCT04711603	CKD-aP	3	Active, not recruiting	172	dose undefined, administered after each dialysis session (3 times/week)	2021-
Study to Evaluate the Efficacy and Safety of Oral Difelikefalin (CR845) for Moderate to Severe Pruritus in Subjects With Notalgia Paresthetica (KOMFORT)	NCT04706975	CKD-aP and notalgia paresthetica	2	Recruiting	120	2.0 mg; oral; twice a day	2021-
A Study to Evaluate the Safety and Efficacy of Difelikefalin in Advanced Chronic Kidney Disease Patients With Moderate-to-Severe Pruritus and Not on Dialysis	NCT05342623	CKD-aP	3	Before recruiting	400	1.0 mg; oral; once a day	2022-
Study to Evaluate the Efficacy and Safety of Oral Difelikefalin (CR845) for Moderate to Severe Pruritus in Subjects With Atopic Dermatitis	NCT04018027	Atopic Dermatitis associated pruritus	2	Completed	401	0.25 mg, 0.5 mg, or 1.0 mg, oral; twice a day	2019–2022
Study to Evaluate the Safety and Efficacy of Oral CR845 (Difelikefalin) in Patients With Primary Biliary Cholangitis (PBC) and Moderate-to-Severe Pruritus	NCT03995212	Cholestatic Pruritus	2	Recruiting	60	1.0 mg; oral; twice a day	2019–2021

## Data Availability

Data sharing not applicable.

## References

[1] Metzger M., Abdel-Rahman E.M., Boykin H., Song M.-K. (2021). A Narrative Review of Management Strategies for Common Symptoms in Advanced CKD. Kidney Int. Rep..

[2] Nair D., Finkelstein F.O. (2020). Pruritus as a Patient-Reported Primary Trial End Point in Hemodialysis: Evaluation and Implications. Am. J. Kidney Dis..

[3] Makar M., Smyth B., Brennan F. (2021). Chronic Kidney Disease-Associated Pruritus: A Review. Kidney Blood Press. Res..

[4] Agarwal P., Garg V., Karagaiah P., Szepietowski J.C., Grabbe S., Goldust M. (2021). Chronic Kidney Disease-Associated Pruritus. Toxins.

[5] Kim D., Pollock C. (2021). Epidemiology and Burden of Chronic Kidney Disease-Associated Pruritus. Clin. Kidney J..

[6] Reszke R., Szepietowski J.C. (2018). End-Stage Renal Disease Chronic Itch and Its Management. Dermatol. Clin..

[7] Verduzco H.A., Shirazian S. (2020). CKD-Associated Pruritus: New Insights Into Diagnosis, Pathogenesis, and Management. Kidney Int. Rep..

[8] Martin C.E., Clotet-Freixas S., Farragher J.F., Hundemer G.L. (2020). Have We Just Scratched the Surface? A Narrative Review of Uremic Pruritus in 2020. Can. J. Kidney Health Dis..

[9] Urbonas A., Schwartz R.A., Szepietowski J.C. (2001). Uremic Pruritus—An Update. Am. J. Nephrol..

[10] Simonsen E., Komenda P., Lerner B., Askin N., Bohm C., Shaw J., Tangri N., Rigatto C. (2017). Treatment of Uremic Pruritus: A Systematic Review. Am. J. Kidney Dis..

[11] Seckin D., Demircay Z., Akin O. (2007). Generalized Pruritus Treated with Narrowband UVB. Int. J. Dermatol..

[12] Gilchrest B.A. (1979). Ultraviolet Phototherapy of Uremic Pruritus. Int. J. Dermatol..

[13] Hsu M.M.L., Yang C.C. (2003). Uraemic Pruritus Responsive to Broadband Ultraviolet (UV) B Therapy Does Not Readily Respond to Narrowband UVB Therapy. Br. J. Dermatol..

[14] Ko M.J., Yang J.Y., Wu H.Y., Hu F.C., Chen S.I., Tsai P.J., Jee S.H., Chiu H.C. (2011). Narrowband Ultraviolet B Phototherapy for Patients with Refractory Uraemic Pruritus: A Randomized Controlled Trial. Br. J. Dermatol..

[15] Mettang T., Kremer A.E. (2015). Uremic Pruritus. Kidney Int..

[16] Reich A., Szepietowski J.C. (2010). Opioid-Induced Pruritus: An Update. Clin. Exp. Dermatol..

[17] Wieczorek A., Krajewski P., Kozioł-Gałczyńska M., Szepietowski J.C. (2020). Opioid Receptors Expression in the Skin of Haemodialysis Patients Suffering from Uraemic Pruritus. J. Eur. Acad. Dermatol. Venereol..

[18] Terg R., Coronel E., Sordá J., Muñoz A.E., Findor J. (2002). Efficacy and Safety of Oral Naltrexone Treatment for Pruritus of Cholestasis, a Crossover, Double Blind, Placebo-Controlled Study. J. Hepatol..

[19] Wolfhagen F.H.J., Sternieri E., Hop W.C.J., Vitale G., Bertolotti M., van Buuren H.R. (1997). Oral Naltrexone Treatment for Cholestatic Pruritus: A Double-Blind, Placebo-Controlled Study. Gastroenterology.

[20] Bergasa N.V., Ailing D.W., Talbot T.L., Swain M.G., Yurdaydin C., Turner M.L., Schmitt J.M., Walker E.C., Jones E.A. (1995). Effects of Naloxone Infusions in Patients with the Pruritus of Cholestasis. A Double-Blind, Randomized, Controlled Trial. Ann. Intern. Med..

[21] Pauli-Magnus C., Mikus G., Alscher D.M., Kirschner T., Nagel W., Gugeler N., Risler T., Berger E.D., Kuhlmann U., Mettang T. (2000). Naltrexone Does Not Relieve Uremic Pruritus: Results of a Randomized, Double-Blind, Placebo-Controlled Crossover Study. J. Am. Soc. Nephrol..

[22] Phan N.Q., Bernhard J.D., Luger T.A., Ständer S. (2010). Antipruritic Treatment with Systemic μ-Opioid Receptor Antagonists: A Review. J. Am. Soc. Nephrol..

[23] Antal A.S., Bernhard J., Ständer S. (2012). Systemic Kappa Opioid Receptor Agonists in the Treatment of Chronic Pruritus: A Literature Review. Acta Derm. Venereol..

[24] Lazenka M.L., Moerke M.J., Townsend E.A., Freeman K.B., Carroll F.I., Negus S.S. (2017). Dissociable Effects of the Kappa Opioid Receptor Agonist Nalfurafine on Pain/Itch-Stimulated and Pain/Itch-Depressed Behaviors in Male Rats. Psychopharmacology.

[25] Jain M.R., Patel R.B., Prajapati K.D., Vyas P., Bandyopadhyay D., Prajapati V., Bahekar R., Patel P.N., Kawade H.M., Kokare D.M. (2022). ZYKR1, a Novel, Potent, and Peripherally Selective Kappa Opioid Receptor Agonist Reduces Visceral Pain and Pruritus in Animal Models. Eur. J. Pharmacol..

[26] Inan S., Dun N.J., Cowan A. (2021). Antipruritic Effect of Nalbuphine, a Kappa Opioid Receptor Agonist, in Mice: A Pan Antipruritic. Molecules.

[27] Togashi Y., Umeuchi H., Okano K., Ando N., Yoshizawa Y., Honda T., Kawamura K., Endoh T., Utsumi J., Kamei J. (2002). Antipruritic Activity of the κ-Opioid Receptor Agonist, TRK-820. Eur. J. Pharmacol..

[28] Nakao K., Hirakata M., Miyamoto Y., Kainoh M., Wakasa Y., Yanagita T. (2016). Nalfurafine Hydrochloride, a Selective κ Opioid Receptor Agonist, Has No Reinforcing Effect on Intravenous Self-Administration in Rhesus Monkeys. J. Pharmacol. Sci..

[29] Ueno Y., Mori A., Yanagita T. (2013). One Year Long-Term Study on Abuse Liability of Nalfurafine in Hemodialysis Patients. Int. J. Clin. Pharmacol. Ther..

[30] Miyamoto Y., Oh T., Aihara E., Ando A. (2022). Clinical Profiles of Nalfurafine Hydrochloride for the Treatment of Pruritus Patients. Handb. Exp. Pharmacol..

[31] Weisshaar E., Szepietowski J.C., Bernhard J.D., Hait H., Legat F.J., Nattkemper L., Reich A., Sadoghi B., Sciascia T.R., Zeidler C. (2022). Efficacy and Safety of Oral Nalbuphine Extended Release in Prurigo Nodularis: Results of a Phase 2 Randomized Controlled Trial with an Open-Label Extension Phase. J. Eur. Acad. Dermatol. Venereol..

[32] Kumagai H., Ebata T., Takamori K., Muramatsu T., Nakamoto H., Suzuki H. (2010). Effect of a Novel Kappa-Receptor Agonist, Nalfurafine Hydrochloride, on Severe Itch in 337 Haemodialysis Patients: A Phase III, Randomized, Double-Blind, Placebo-Controlled Study. Nephrol. Dial. Transplant..

[33] Pilla J.E., Devulapally P. (2021). Difelikefalin.

[34] Shirazian S., Spencer R., Kilfeather S. (2022). Reduction of Pruritus by Difelikefalin Correlates With Reductions in Markers for Pruritus and Inflammation in Subjects Undergoing Hemodialysis. Am. J. Kidney Dis..

[35] Viscusi E.R., Torjman M.C., Munera C.L., Stauffer J.W., Setnik B.S., Bagal S.N. (2021). Effect of Difelikefalin, a Selective Kappa Opioid Receptor Agonist, on Respiratory Depression: A Randomized, Double-Blind, Placebo-Controlled Trial. J. Clin. Transl. Sci..

[36] Shram M.J., Spencer R.H., Qian J., Munera C.L., Lewis M.E., Henningfield J.E., Webster L., Menzaghi F. (2022). Evaluation of the Abuse Potential of Difelikefalin, a Selective Kappa-Opioid Receptor Agonist, in Recreational Polydrug Users. J. Clin. Transl. Sci..

[37] Deeks E.D. (2021). Difelikefalin: First Approval. Drugs.

[38] Page M.J., McKenzie J.E., Bossuyt P.M., Boutron I., Hoffmann T.C., Mulrow C.D., Shamseer L., Tetzlaff J.M., Akl E.A., Brennan S.E. (2021). The PRISMA 2020 Statement: An Updated Guideline for Reporting Systematic Reviews. BMJ.

[39] Fishbane S., Mathur V., Germain M.J., Shirazian S., Bhaduri S., Munera C., Spencer R.H., Menzaghi F., Aaronson M., Alford K. (2020). Randomized Controlled Trial of Difelikefalin for Chronic Pruritus in Hemodialysis Patients. Kidney Int. Rep..

[40] Fishbane S., Jamal A., Munera C., Wen W., Menzaghi F. (2020). A Phase 3 Trial of Difelikefalin in Hemodialysis Patients with Pruritus. N. Engl. J. Med..

[41] Narita I., Tsubakihara Y., Uchiyama T., Okamura S., Oya N., Takahashi N., Gejyo F., Yamamoto A., Ichikawa A., Ohishi A. (2022). Efficacy and Safety of Difelikefalin in Japanese Patients With Moderate to Severe Pruritus Receiving Hemodialysis: A Randomized Clinical Trial. JAMA Netw. Open.

[42] McGuinness L.A., Higgins J.P.T. (2020). Risk-of-bias VISualization (Robvis): An R Package and Shiny Web App for Visualizing Risk-of-bias Assessments. Res. Syn. Meth..

[43] Hashimoto T., Yosipovitch G. (2019). Itching as a Systemic Disease. J. Allergy Clin. Immunol..

[44] Kimata N., Fuller D.S., Saito A., Akizawa T., Fukuhara S., Pisoni R.L., Robinson B.M., Akiba T. (2014). Pruritus in Hemodialysis Patients: Results from the Japanese Dialysis Outcomes and Practice Patterns Study (JDOPPS). Hemodial. Int..

[45] Rayner H.C., Larkina M., Wang M., Graham-Brown M., van der Veer S.N., Ecder T., Hasegawa T., Kleophas W., Bieber B.A., Tentori F. (2017). International Comparisons of Prevalence, Awareness, and Treatment of Pruritus in People on Hemodialysis. Clin. J. Am. Soc. Nephrol. CJASN.

[46] Lai J.W., Chen H.C., Chou C.Y., Yen H.R., Li T.C., Sun M.F., Chang H.H., Huang C.C., Tsai F.J., Tschen J. (2017). Transformation of 5-D Itch Scale and Numerical Rating Scale in Chronic Hemodialysis Patients. BMC Nephrol..

[47] Vernon M.K., Swett L.L., Speck R.M., Munera C., Spencer R.H., Wen W., Menzaghi F. (2021). Psychometric Validation and Meaningful Change Thresholds of the Worst Itching Intensity Numerical Rating Scale for Assessing Itch in Patients with Chronic Kidney Disease-Associated Pruritus. J. Patient-Rep. Outcomes.

[48] Kimel M., Zeidler C., Kwon P., Revicki D., Ständer S. (2020). Validation of Psychometric Properties of the Itch Numeric Rating Scale for Pruritus Associated With Prurigo Nodularis: A Secondary Analysis of a Randomized Clinical Trial. JAMA Dermatol..

[49] Naegeli A.N., Flood E., Tucker J., Devlen J., Edson-Heredia E. (2015). The Worst Itch Numeric Rating Scale for Patients with Moderate to Severe Plaque Psoriasis or Psoriatic Arthritis. Int. J. Dermatol..

[50] Elman S., Hynan L.S., Gabriel V., Mayo M.J. (2010). The 5-D Itch Scale: A New Measure of Pruritus. Br. J. Dermatol..

[51] Mathur V.S., Lindberg J., Germain M., Block G., Tumlin J., Smith M., Grewal M., McGuire D. (2010). A Longitudinal Study of Uremic Pruritus in Hemodialysis Patients. Clin. J. Am. Soc. Nephrol. CJASN.

[52] Cheung H.N., Chan Y.S., Hsiung N.H. (2021). Validation of the 5-D Itch Scale in Three Ethnic Groups and Exploring Optimal Cutoff Values Using the Itch Numerical Rating Scale. Biomed. Res. Int..

[53] A Phase III Clinical Study of MR13A9 in Hemodialysis Patients with Pruritus. https://www.clinicaltrials.gov/ct2/show/NCT04711603?term=mr13a9&draw=2&rank=1.

[54] A Clinical Study of MR13A9 in Hemodialysis Patients with Pruritus. https://www.clinicaltrials.gov/ct2/show/NCT03802617?term=mr13a9&draw=2&rank=2.

[55] CR845-CLIN3103: A Global Study to Evaluate the Safety and Efficacy of CR845 in Hemodialysis Patients with Moderate-to-Severe Pruritus. https://www.clinicaltrials.gov/ct2/show/NCT03636269?term=difelikefalin&draw=2&rank=10.

[56] Extension Study to Evaluate IV CR845 in Hemodialysis Patients with Moderate-to-Severe Pruritus. https://www.clinicaltrials.gov/ct2/show/NCT03281538?term=difelikefalin&draw=2&rank=14.

[57] Study to Evaluate IV CR845 in Hemodialysis Patients with Moderate-to-Severe Pruritus. https://www.clinicaltrials.gov/ct2/show/NCT02858726?term=difelikefalin&draw=2&rank=13.

[58] A Study to Evaluate the Safety and Efficacy of CR845 in Hemodialysis Patients with Moderate-to-Severe Pruritus (KALM-1). https://www.clinicaltrials.gov/ct2/show/NCT03422653?term=difelikefalin&draw=2&rank=12.

[59] CR845-CLIN3105: A Study to Evaluate the Safety and Effectiveness of CR845 in Hemodialysis Patients with Moderate-to-Severe Pruritus. https://www.clinicaltrials.gov/ct2/show/NCT03998163?term=difelikefalin&draw=2&rank=11.

[60] A Study to Evaluate the Safety and Efficacy of CR845 in Chronic Kidney Disease Patients with Moderate-to-Severe Pruritus. https://www.clinicaltrials.gov/ct2/show/NCT03617536?term=difelikefalin&draw=2&rank=9.

[61] Study to Evaluate the Pharmacokinetics and Metabolism of [14C] CR845 (Difelikefalin) in Patients with End Stage Renal Disease on Hemodialysis and in Healthy Subjects. https://www.clinicaltrials.gov/ct2/show/NCT03947970?term=difelikefalin&draw=2&rank=8.

[62] Intermediate-Size Patient Population Expanded Access Program for Intravenous Difelikefalin. https://www.clinicaltrials.gov/ct2/show/NCT05031546?term=difelikefalin&draw=2&rank=4.

[63] A Study to Evaluate the Safety and Efficacy of Difelikefalin in Advanced Chronic Kidney Disease Patients with Moderate-to-Severe Pruritus and Not on Dialysis. https://www.clinicaltrials.gov/ct2/show/NCT05342623?term=difelikefalin&draw=2&rank=2.

[64] Study to Evaluate the Efficacy and Safety of Oral Difelikefalin (CR845) for Moderate to Severe Pruritus in Subjects with Notalgia Paresthetica (KOMFORT). https://www.clinicaltrials.gov/ct2/show/NCT04706975?term=difelikefalin&draw=2&rank=1.

[65] Study to Evaluate the Safety and Efficacy of Oral CR845 (Difelikefalin) in Patients with Primary Biliary Cholangitis (PBC) and Moderate-to-Severe Pruritus. https://www.clinicaltrials.gov/ct2/show/NCT03995212?term=difelikefalin&draw=2&rank=7.

[66] Study to Evaluate the Efficacy and Safety of Oral Difelikefalin (CR845) for Moderate to Severe Pruritus in Subjects with Atopic Dermatitis. https://www.clinicaltrials.gov/ct2/show/NCT04018027?term=difelikefalin&draw=2&rank=5.

[67] Home-ClinicalTrials.Gov. https://www.clinicaltrials.gov/.

[68] Little P.J. (2013). Peripherally Restricted Opioid Analgesics. ACS Symp. Ser..

[69] Fishbane S., Wen W., Munera C., Menzaghi F. (2021). Long-Term Safety and Efficacy of Difelikefalin in Patients With Chronic Kidney Disease–Associated Pruritus: Analysis From KALM-1 and KALM-2. Am. J. Kidney Dis..

[70] Kapruvia|European Medicines Agency. https://www.ema.europa.eu/en/medicines/human/EPAR/kapruvia.

[71] Harden R., Burns J., Connolly S., Kirsling A., Abousaad E., Khoury A., Walega D. (2015). (422) CR845, a Peripheral Kappa Opioid, Provides Better Pain Relief with Less Nausea and Vomiting than Placebo in Patients after Bunionectomy. J. Pain.

